# Complementary and alternative medicine (CAM) and atopic eczema 

**DOI:** 10.5414/ALX01287E

**Published:** 2017-08-04

**Authors:** T. Schäfer

**Affiliations:** Ratekau, Germany

**Keywords:** atopic eczema, evidence-based medicine, complementary alternative medicine

## Abstract

Abstract. There is a substantial and growing interest in complementary and alternative medicine (CAM) in the general population. This paper aims to answer in how far patients with atopic eczema use CAM and which techniques. Furthermore the evidence basis on the efficacy of CAM in the use for atopic eczema should be reviewed. For that purpose randomized controlled trials (RCT) were searched systematically. In Germany about 46% of the general population and up to 51% of inpatients with eczema use CAM. Acupuncture, homeopathy, diets and supplements comprise the most popular techniques. Better educated, middle-aged women use CAM more frequently. In general the evidence basis concerning studies on the efficacy (and safety) of CAM for atopic eczema with appropriate size and quality is limited. Most studies were found on essential fatty acids and Chinese herbs, whereby the results remain conflicting. There was not enough evidence to assess the efficacy of acupuncture, homeopathy and salt baths. A single study on bioresonance did not show superiority compared to a sham procedure. Single studies indicated beneficial effects for topical hypericum, autologous blood injection, massage therapy, Vitamin E and D, and topical Viatmin B12. These results must be confirmed by future studies. CAM are frequently used in atopic eczema, the evidence basis for that, however, is limited.

German version published in Allergologie, Vol. 33, No. 4/2010, pp. 180-189

There is evidence for a growing interest in so-called complementary and alternative medicine (CAM) as a treatment of atopic eczema [1, 2, 3]. In this review we are trying to present the published evidence in terms of a safety and efficacy confirmation for these methods. 

For this purpuse the electronic database PubMed was systematically searched for randomized controlled trials on the treatment of atopic eczema using CAM which had been published until October 2009. Search terms included “atopic dermatitis or eczema” and “complementary or alternative medicine” as well as individual procedures such as “acupuncture, homeopathy, bioresonance, phytotherapy”. 

The underlying ideas and concepts concerning effects and modes of application cannot be presented in detail here. The interested reader may refer to further literature [4]. 

## Definition 

“Complementary medicine is diagnosis, treatment and/or prevention which complements mainstream medicine by contributing to a common whole, by satisfying a demand not met by orthodoxy or by diversifying the conceptual frameworks of medicine” [5]. 

## Use in the general population 

**Figure 1.**
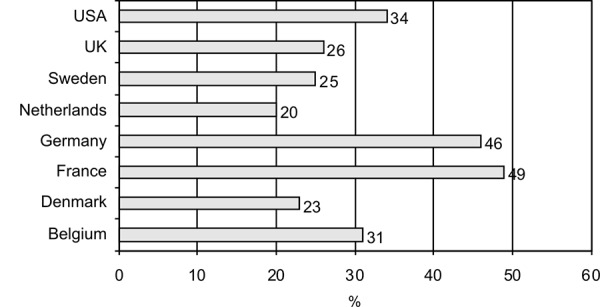
Prevalence of population-related use of complementary and alternative medicine in the USA and selected European countries [6].

Within Europe the demand for CAM seems to be highest in France (49%) and Germany (46%) (Figure 1) [6]. These high percentages in France and Germany were confirmed by a second cross-sectional study published in 2000 in which the prevalence was 65% in Germany and 49% in France [2]. The growing interest in CAM in the general population was confirmed by a US study showing that the use of complementary and alternative medicine increased from 33.8% to 42.1% in the years 1990 – 1997 [7]. 

Application of complementary and alternative medicine in cases of atopic eczema 

Only few studies have investigated the usage patterns of CAM in cases of atopic eczema or similar diseases. In a Swiss study 202 patients of a rehabilitation hospital suffering from atopic eczema or inhalant allergies were investigated. Of these patients, 37% declared that they had been using CAM before. The most frequently used methods were homeopathy (48%), diets (35%), herbs (28%), autologous blood injection (28%), phytotherapy (20%) and acupuncture (18%) [8]. 

In a Norwegian study 444 hospitalized patients with atopic eczema were interviewed and 51% reported previous experience with CAM. The most popular procedures were homeopathy (34%), herbs (19%), dietary supplementation (18%), diets (18%) and acupuncture (11%) [9]. 

According to population-based telephone interviews with 351 adult allergy patients 26.5% of the interviewees reported to have used CAM at least once [10]. The most frequently used methods were homeopathy (35.3%), autologous blood injection (28.1%), acupuncture (16.6%) and bioresonance (10.0%). 

## Application of complementary and alternative medicine by dermatologists 

A British health services research study compared the treatment patterns of dermatologists in Japan, the USA and the UK [11]. According to this study the dermatologists who most frequently applied CAM were the Japanese (27%). Interestingly, British and American dermatologists who were older than 45 years applied CAM significantly more frequently while this relationship was reversed in the Japanese dermatologists. 

## Specific complementary and alternative medicine 

### Essential fatty acids 

Concerning polyunsaturated fatty acids (PUFA) there has to be a differentiation regarding omega-3 fatty acids like eicosapentaenoic acid and their metabolites on the one hand and omega-6 acids like gamma-linolenic and arachidonic acid and their metabolites on the other. The supplementation with omega-3 fatty acids is based on the assumption that the inflammatory profile of omega-6 fatty acids and their metabolites is higher than that of omega-3 fatty acids and their metabolites and that by supplementation the metabolism can be shifted towards less inflammatory metabolites. The oral and topical application of omega-3 fatty acids was investigated in patients with atopic eczema. The most common preparations are eicosapentaenoic acid, seed oil of evening primrose (Epogam), borage oil and fish oil. The health technology assessment report on the treatment of atopic eczema published in 2000 summarizes all available randomized controlled trials on the supplementation with essential fatty acids [12]. The authors describe a meta-analysis with nine RCTs [13] and a large study by Bamford et al. [14]. The meta-analysis concludes that seed oil of evening primrose has a moderate beneficial effect. The results of the two largest and well-documented studies did not show superiority to placebo. The results of the other nine published RCTs were inconsistent. In a clinical study carried out by Ring and Kunz [15] 17 patients were treated with either eicosapentaenoic acid or placebo for 3 months. At the end of the study the clinical parameters had significantly improved in both groups with no inter-group differences being observed. In an Indian study significant therapeutic effects of 500 mg seed oil of evening primrose compared to 300 mg sunflower oil, both applied as capsules for a duration of 5 months, could be shown [16]. Further meta-analyses and systematic reviews with regard to seed oil of evening primrose and gamma-linolenic acid (GLA) are expected. 

Apart from four smaller studies [17, 18, 19, 20] with inconsistent results there are only two large RCTs on borage oil in atopic eczema. In the investigation by Henz et al. [21] 160 adult patients were treated with either borage oil capsules or placebo for 24 weeks. No differences were observed with regard to corticosteroid use. In a subgroup analysis concerning the increase of the erythrocyte dihomolinolenic acid significant differences in favor of borage oil supplementation were observed. This might suggest a beneficial effect in patients who absorb and metabolize gamma-linolenic acid and warrants further investigation. In another British study a total of 140 patients, including 69 children, were treated with either borage oil or placebo for 12 weeks [22]. No significant group differences were observed for the assessed parameters (severity, symptoms, overall assessment, drug use) [22]. 

A German study compared the daily administration of 5.4 docosahexaenic acid (DHA) in 21 evaluable patients with the administration of an energetically equal amount of saturated fatty acids in 23 patients for a duration of 8 weeks. Although the SCORAD in the DHA group reduced significantly, the inter-group comparison was not statistically significant [23]. 

In a comparative investigation on dietary hemp seed and olive oil the skin physiological parameters and symptoms improved under hemp oil treatment [24]; there were, however, obviously no statistically significant differences to the control group [24]. 

A more recent RCT in 20 in-patients with atopic eczema compared fish oil and soy bean oil infusions and showed marked clinical improvements in both groups with significantly better results in the group treated with fish oil [25]. Some smaller RCTs have also shown beneficial effects [26, 27, 28], although the largest well-documented study did not show any difference between fish oil and placebo [29]. 

Seed oil of evening primrose is also used for topical treatment. Although a pilot study could show beneficial effects [30], subsequent studies could not establish a dose-effect relationship [31]. Other studies failed to demonstrate a beneficial effect on the skin barrier function [32]. Larger studies on this topic are lacking. 

### Topical phytotherapy 

For a long time herbal formulations have been used internally or externally for skin diseases, mainly due to their anti-inflammatory and antipruritic effects. The backgrounds of phytotherapy in dermatology have been summarized in a review [33]. Concerning topical application, two RCTs that analyzed the safety and efficacy of a camomile preparation and a St. John’s wort preparation, respectively, could be found [34, 35]. In a side-by-side trial in 69 patients with atopic eczema the commercial camomile extract preparation (Kamillosan-Creme) was compared with either a 0.5% hydrocortisone cream or a foundation cream that did not contain an active agent. With regard to the most important clinical endpoints pruritus, erythema and desquamation the camomile preparation was slightly superior to the hydrocortisone preparation after 2 weeks. There was, however, no difference between the camomile preparation and the foundation cream. Unfortunately, the publication does not give information on the results of statistical tests. In a side-by-side study in 18 patients with weak-to-moderate atopic eczema the cream containing St. John’s wort extract was standardized to a hyperforin content of 1.5% and was compared to the vehicle cream. After 4 weeks the modified SCORAD index had improved under both treatments with the improvements being significantly more profound in the active group. These beneficial results should be proven in larger studies and in comparison to the standard therapy. 

A further study compared the topical preparation of pansy, pennywort and holly with a vehicle cream in 88 patients and no significant differences were observed. In a subgroup analysis a statistically significant superiority of the herbal preparation under cool and dry climatic conditions was observed. 

Generally, herbal extracts can cause contact sensitization and subsequent contact allergy. This has been intensely investigated and confirmed by numerous clinical reports [37, 38]. It could also be shown that so-called phytocosmetic creams contain a mixture of herbal extracts which contain, among others, triamcinolonacetonide as an active substance [39]. 

### Chinese herbs 

Chinese herbs are part of Traditional Chinese Medicine (TCM) which consists of topically or orally administered Chinese herbs, acupuncture, diet and physical exercise [40]. Chinese herbs are available for the treatment of atopic eczema, either for oral application or as an extract that normally consists of 10 different herbs. The first non-Chinese randomized controlled trial on the use of Chinese herbs for the treatment of atopic eczema was published in 1992 by Sheehan et al. [41] and later included in a systematic review [42]. In a cross-over design 37 children and 31 adults were treated with either an active herbal mixture or a placebo for 8 weeks. The severity index included erythematous skin lesions and the percentage of involved skin surface. In the children the percentaged decrease of skin lesions was 63.1% in the active group and 6.2% in the placebo group. After 1 year 23 children who had continued the treatment with Chinese herbs showed better results than those children who had discontinued the treatment [43]. In the adults the geometric mean for skin surface lesions was 11.3 at the end of the treatment with Chinese herbs, while it was 111 in the placebo group [44]. After 1 year the clinical severity score had reduced by more than 90% in patients who had continued treatment, which was significantly more than in the 11 patients who had not continued treatment. No short-term toxicity was observed in these studies. Nevertheless, routine examinations of the kidney and liver functions as well as of the blood count at the beginning of treatment was recommended. After the examinations, independent investigators described severe side effects including 1 case of fatal hepatitis [45, 46, 47]. 

In a further study a commercially available Chinese herb product (Zemaphyte) was investigated with regard to immunologic target values, and evidence for relevant immunologic and clinical effects was observed [48]. Subsequently, Zemaphyte was compared to placebo in a cross-over design in 37 patients [49]. In both groups a tendency towards clinical improvement was observed and there were no differences between the groups. 

In a recent double-blind placebo-controlled study from Hong Kong 85 children were treated with Chinese herbs in capsule form for 12 weeks. While the SCORAD was reduced significantly in both groups, with the effect being more pronounced in the placebo group, only the verum group showed a significant improvement concerning health-related quality of life and cortisone saving [50]. 

The placebo-controlled oral administration of a herbal extract mixture of yarrow, deadnettle and eleuthero did not show an improvement compared to placebo after 2 weeks [51]. 

Although some reports suggest beneficial effects of the treatment of atopic eczema with Chinese herbs, other studies do not support these observations. It was not at least due to potential side effects that the use of Chinese herbs was not investigated more profoundly. 

### Acupuncture 

Acupuncture belongs to the three CAM therapies that are most frequently applied in cases of allergy [2]. Particularly with regard to asthma there are a series of studies that investigated the safety and efficacy of acupuncture. Although single studies suggest a beneficial effect, the corresponding meta-analysis did not show a significant and clinically relevant effect of acupuncture on asthma [52]. Acupuncture as a treatment of atopic eczema has not been investigated systematically or in the framework of randomized controlled trials. Case series in patients, including patients with atopic eczema, suggest beneficial effects, but studies applying conclusive and consistent methods are lacking [53]. 

### Autologous blood injection 

In many countries autologous blood injection as a CAM is making a comeback. In Germany autologous blood injection amounts to 28% of all CAM treatments in allergy patients and thus ranks second behind homeopathy (35%) [10]. The underlying concept is the expectation that the normally intramuscular re-injection of an autologous blood sample provokes a beneficial immunostimulation. The blood sample can be modified, e.g., using ozone, irradiation or homeopathic substances. There is one RCT that compared the re-injection of 1 – 3 ml of an autologous blood sample with the injection of an identical amount of sterile saline solution over a period of 5 weeks [54]. The patients were recruited via the media and finally 30 persons participated. During the 9-week observation phase the severity index SASSAD was significantly reduced in the verum group (from 23.2 to 10.5), while in the placebo group no change was observed (21.0 vs. 22.5). No significant differences were observed concerning the health-related quality of life and the subjective evaluation of pruritus, skin symptoms and quality of sleep. The results of this study should be verified in larger studies and in different treatment contexts. 

### Bioresonance 

The concept of bioresonance is based on the assumption that a person diseases when electromagnetic frequencies or electric fields in his or her body get out of balance. The users assume that these disturbances in balance modify the chemical composition of the human body. They further believe that these disturbances can be corrected by administering electric energy from outside the body, normally using electronic devices. Until now, one RCT has been published in which the application of bioresonance was compared to a placebo procedure in 36 children with atopic eczema in a specialized rehabilitation center in Davos, Switzerland [55]. After 4 weeks the severity score had improved in both groups, with a slight superiority being present in the active group (difference 12.5 vs. 8.7). There were no statistically significant differences between the groups. Although small effects cannot be excluded, no substantial clinical effect could be shown in this study and further studies including representative outpatients are required. 

### Homeopathy 

In summary, homeopathic treatment according to Hahnemann is based on the idea that large amounts of a substance cause symptoms, while smaller amounts of the same substance are able to cure these symptoms. Homeopathy is frequently used as a CAM treatment in atopic eczema. Larger patients series that were supposed to emphasize the therapeutic effect were published in scientific journals and books [56]. A recent, uncontrolled Japanese study in 17 patients with chronic atopic eczema showed a marked clinical improvement under homeopathic treatment [57]. In Germany, a classical placebo-controlled randomized trial in 60 patients was initiated [58]. The preliminary results do not show superiority of homeopathic treatment; the final results are not yet published. 

### Massage treatment and aromatherapy 

In a randomized study in 20 children the effects of concomitant massage therapy (20 minutes daily for 1 month) was compared with standard therapy alone [59]. The parents of children in the active group reported marked improvements concerning the children’s anxiety, tactile defensiveness and ability to cope with the disease. Furthermore, clinical signs like desquamation and excoriation improved significantly in the massage group. Appropriate statistical comparisons between the groups were, however, not carried out. A further small-scale cross-over study in 8 children compared massage with essential oils (aromatherapy) with conventional massage [3]. Significant improvements were observed in both groups, without any differences between the groups. In view of the small number of cases it is almost impossible to draw any conclusions about a possible beneficial effect of concomitant aromatherapy. 

### Salt water baths 

For a long time salt water baths have been used in cases of chronic inflammatory skin diseases, particularly in psoriasis. Based on this experience and anecdotal evidence salt water baths have recently also been recommended for the treatment of atopic eczema. The efficacy of salt water baths as a single treatment for atopic eczema has not yet been investigated in systematic studies. In current studies salt water baths either formed part of a complex climate therapy or were used in combination with UV irradiation [60, 61]. In a clinical observation of 1,408 patients with atopic eczema who spent 4 – 6 weeks at the Dead Sea a complete remission of the lesions could be seen in 90% of the patients [62]. In another study at the Dead Sea 56 patients with atopic eczema were treated with diluted Dead Sea water or fresh water for 20 minutes twice daily as part of a climate therapeutic regimen. As a result the severity score improved significantly in both groups and no statistically relevant differences were observed between the groups. In another, uncontrolled trial the combination of narrow-spectrum UVB and bathing in solution of Dead Sea salt was investigated. Significant improvements in the SCORAD score were observed in the per-protocol analysis as well as in the intention-to-treat analysis [64]. In a small-scale German study 12 patients were treated with UVA monotherapy and compared with 16 patients who received concomitant baths in salt water [65]. After 20 treatments the SCORAD score had improved significantly in the group that was treated with balneophototheray and only marginally in the group that was treated with irradiation alone. The patients had, however, not been randomized in this small study and the eczema severity scores at baseline suggest that the SCORAD was markedly higher in the patients in the combination therapy group. In another German study balneophototherapy with Dead Sea salt was compared with salt baths alone [66]. Unfortunately, the results for the 8 patients with atopic eczema have not been reported separately. In a randomized Japanese study 100 patients were treated either with sea water or by spraying physiologic saline solution on the skin for 10 minutes daily over a period of 1 week [67]. The clinical improvements were marginal and did not differ statistically. Currently, there is not sufficient evidence from randomized controlled trials that would support the recommendation to treat atopic eczema with salt water baths. 

### Oral vitamins and mineral nutrients 

A total of 6 studies using vitamins or mineral nutrients for the treatment of atopic eczema was identified [68, 69, 70, 71, 72, 73]. In a recent Italian study 96 patients were randomized to either receive 400 IU vitamin E (administered once daily) or placebo over a period of 8 months [72]. According to the subjective assessment of clinical target values after 12 months marked intergroup differences were observed. 46% of patients in the vitamin E group, but only 2% of patients in the placebo group reported a marked improvement. Aggravation was reported by 87% of patients in the placebo group and by 8% of patients in the vitamin E group. Unfortunately, no results of statistical tests have been published. Similarly, a small-scale study in 49 patients showed that the combined administration of vitamin E and vitamin B2 is superior to the administration of vitamin E or vitamin B2 alone with regard to the overall benefit of the overall assessment evaluated by a physician [70]. 

In a further study in 60 adult patients with atopic eczema the combined administration of selenium and vitamin E was compared with selenium administration alone or placebo, respectively, over a period of 12 weeks [69]. The severity score decreased in all three studies without significant differences being observed. In a Hungarian study the administration of multivitamins in 2,090 pregnant women was compared with the administration of trace elements in 2,032 pregnant women over a period of 17 months [68]. The incidence of atopic eczema was slightly higher in the multivitamin group (0.7% vs. 0.2%). Although the authors suggest that this unexpected result could be coincidence, further studies with a prospective approach are required for verification. 

A further small-scale study compared zinc supplementation with placebo in 15 children over a period of 2 months [74]. The severity score decreased in both groups without significant differences being observed. 

In a published RCT pyridoxine (vitamin B6) was compared with placebo in 41 children over a period of 4 weeks [71]. In the pyridoxine group the severity score increased while an improvement could be observed in the placebo group. The differences were not statistically significant. 

In the only pilot study on vitamin D that has so far been carried out 1,000 IU vitamin D were administered to 5 children over a period of 1 month. The comparison with 6 control children showed a superiority in the EASI score which was, however, not statistically significantly [73]. 

There is preliminary evidence that suggests that vitamins, in particular vitamin E and D, might have a beneficial influence on atopic eczema, but further studies are necessary to verify these results. 

### Topical vitamin B12 

There are two smaller studies with side-by-side trials which suggest that a topical preparation of 0.07% vitamin B12 based on avocado oil has a beneficial effect on atopic eczema as compared to placebo. After an 8-week application period the decrease in the modified SASSAD score of 41 adults was more pronounced in the areas treated with verum than in the placebo-treated region. The overall evaluation of patients and investigators also showed a significant superiority of the verum in this German study [75]. In the USA the preparation was tested in a similar design in 21 children and also in this study the SCORAD showed a significant superiority of the verum [76]. Larger studies should be carried out in order to verify these results. 

### Side effects 

In contrast to the popular opinion complementary and alternative medicine is not free of side effects. Strict and frequently absurd dietary restrictions can cause malnutrition. Therapeutic procedures that use organic material from animals and plants can be accompanied by severe allergic or toxic reactions.
